# First Observations of a Deep‐Water Seagrass Meadow (*Thalassodendron ciliatum*) on an Oceanic Reef in the Southern Coral Sea Marine Park, Australia

**DOI:** 10.1002/ece3.71254

**Published:** 2025-04-17

**Authors:** G. F. Galbraith, B. J. Cresswell, M. Russell, A. S. Hoey

**Affiliations:** ^1^ Marine Biology and Aquaculture, College of Science and Engineering James Cook University Townsville Queensland Australia; ^2^ ARC Centre of Excellence for Coral Reef Studies James Cook University Townsville Queensland Australia; ^3^ AIMS@JCU Australian Institute of Marine Science Townsville Queensland Australia; ^4^ Parks Australia Brisbane Queensland Australia

**Keywords:** coral reef fishes, habitat heterogeneity, lagoon bommies, marine biodiversity, remotely operated vehicle

## Abstract

Tropical seagrass meadows are important global marine ecosystems that provide critical ecosystem goods and services. The extent of global seagrass meadows is mostly mapped from shallow coastal regions and not well known or sampled from deeper offshore locations. Seagrasses can, however, form deep‐water meadows, which likely significantly increase the total area of global seagrass ecosystems and may contribute important ecological functions to offshore tropical seascapes. Here we report the first observation of a dense meadow of *Thalassodendron ciliatum* at a depth of 25 m using remotely operated vehicles (ROVs) from the Coral Sea Marine Park (CSMP). Despite significant survey effort in the region, to date there have only been three other observations of seagrass in the CSMP, all sparse and small patches of *Halophila ovalis* and *Halophila decipiens* . We discuss the significance of this newly discovered meadow within the context of current reef health monitoring of the CSMP, reef fish biodiversity and the ecological value of deep‐water seagrass habitats for offshore coral reef systems like the Coral Sea.

## Graphical abstract

First record of a significant deep‐water seagrass meadow in the Coral Sea Marine Park and observations on associated reef fishes.

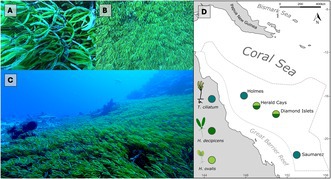

## 1 Introduction

Tropical seagrasses are typically found in shallow coastal marine and estuarine environments where they grow in soft sediments, rely on adequate light and can form dense meadows (Larkum et al. 2006; Unsworth and Cullen‐Unsworth 2017). Seagrass meadows support many important ecological functions, including as a food source for grazing species (e.g., turtles and dugongs; Aragones and Marsh 1999), and indirectly through the provision of shelter and nursery grounds for numerous marine taxa (Duffy 2006). In particular, seagrass meadows often support a high diversity and abundance of juvenile fishes (Nagelkerken et al. 2000; Heck Jr et al. 2003), providing an important link between fish populations in other marine habitats (e.g., coral reefs) which, in turn, support fisheries at both small and local scales (Unsworth et al. 2019; Sambrook et al. 2019; Sievers et al. 2020). Seagrass meadows are also key contributors to physical processes, including substrate stabilisation (Christianen et al. 2013), nutrient filtration (Lee and Dunton 1999), maintenance of water quality (Lamb et al. 2017), storm protection (James et al. 2021) and are increasingly recognised as a valuable global carbon sink (Fourqurean et al. 2012). These have all led to seagrasses being considered the third most valuable ecosystem per unit area (Costanza et al. 1997; Barbier et al. 2011; Unsworth and Cullen‐Unsworth 2014).

There are 72 extant species of seagrass globally, of which 15 species from 9 genera have known distributions on the east coast of Australia and Great Barrier Reef (GBR) (Lee Long et al. 1993; Carter, Collier, et al. 2021). All are mostly reported from depths of less than 20 m but have been recorded as deep as 76 m from the GBR (Carter, McKenna, et al. 2021). Significant efforts have mapped much of the extent of seagrass meadows in shallow coastal waters along Australia's east coast (Carter, McKenna, et al. 2021; Carter et al. 2024; Coles et al. 2016). However, deeper offshore locations in the region are not well sampled and globally relatively little is known about the distribution of deeper (> 20 m) offshore seagrasses compared to their shallow inshore counterparts (Coles et al. 2009; York et al. 2015; Esteban et al. 2018).

**FIGURE 1:**
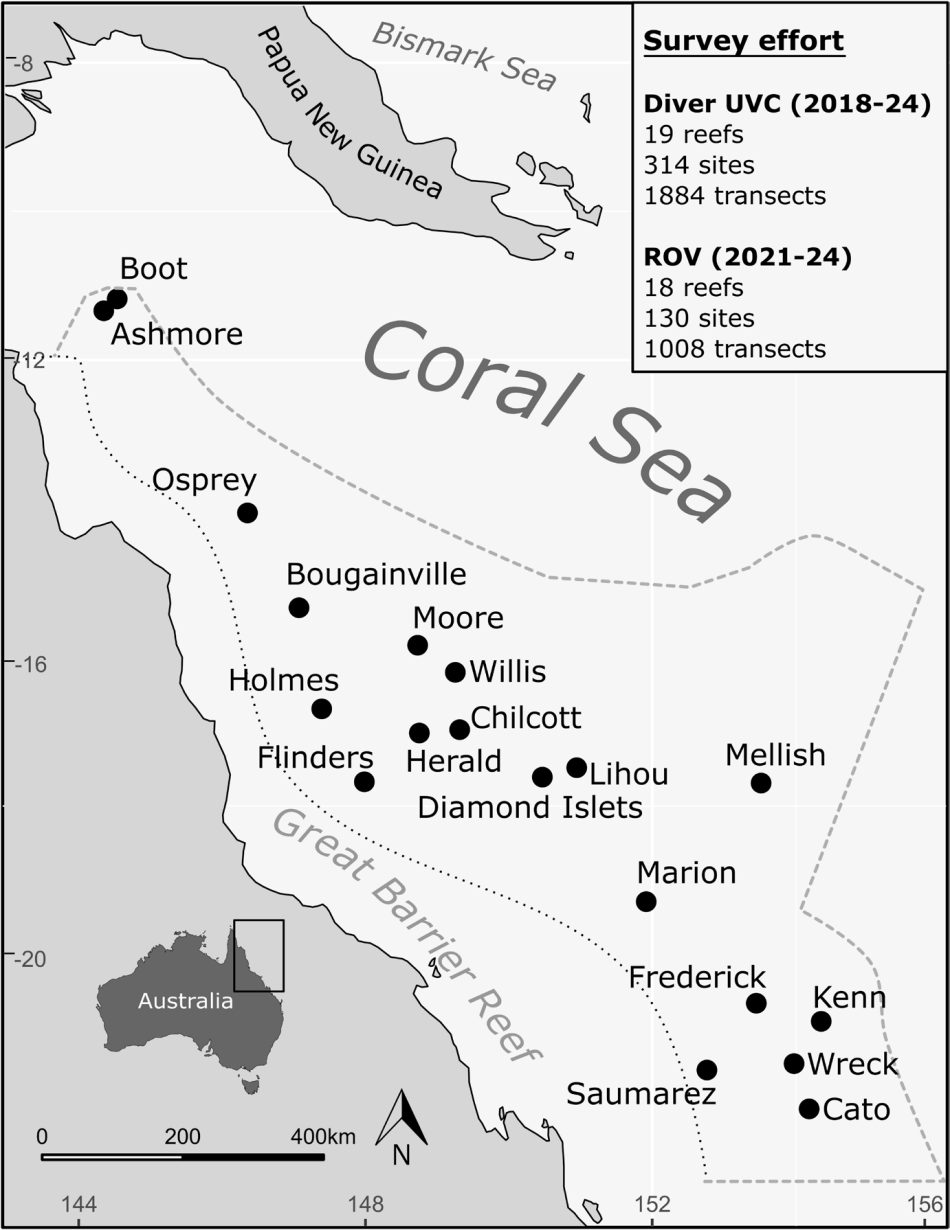
Location of the Coral Sea Marine Park (CSMP), offshore from the Great Barrier Reef, east coast Australia. Inset in the top right‐hand corner details survey effort conducted by diver underwater visual census and remotely operated vehicles between 2018 and 2024 at 448 sites at 19 reefs throughout the CSMP at depths between 2 and 110 m (annual monitoring by James Cook University).

Australia's Coral Sea Marine Park (CSMP) is 989,836km^2^ and situated off Australia's north‐east coast; bounded by Papua New Guinea to the north, the Solomon Islands, Vanuatu and New Caledonia to the east, Australia's Great Barrier Reef to the west and the Tasman Sea to the south (Figure 1). The region is characterised by isolated seamounts rising from four major deep‐water plateaus that are separated from each other and from neighbouring continental shelves by deep (up to 4000 m) oceanic water (Beaman 2010). These seamounts possess diverse marine habitats including shallow coral reefs, their associated lagoons, coral bommies and deep mesophotic to rariphotic coral ecosystems (Ceccarelli 2011; Ceccarelli et al. 2013; Muir et al. 2015; Englebert et al. 2017; Baldwin et al. 2018). Recent ecological monitoring and baseline surveys have begun to establish knowledge of shallow coral reefs in the CSMP (Ayling and Ayling 1985; Ceccarelli et al. 2008; Hoey et al. 2023), but much of the region's biodiversity (especially in deeper waters; Beaman et al. 2016; Bridge et al. 2019; Galbraith, Cresswell, et al. 2022; Galbraith et al. 2024) remains poorly documented. Specifically, the occurrence and distribution of tropical seagrasses in the CSMP are largely unknown.

Between 2018 and 2024, annual reef health monitoring surveys were conducted at 19 reefs in the CSMP (Hoey et al. 2020, 2021, 2023). This survey effort constituted 1884 diver underwater visual census (UVC) transects (50 m) across 314 sites conducted at depths between 2 and 10 m. To access deeper coral reef habitats in the CSMP, remotely operated vehicles (ROVs) were utilised from 2021 to conduct concurrent monitoring and exploration of CSMP reefs below depths attainable by diver‐based UVC (10—110 m). ROV survey effort between 2021 and 2024 comprised 1008 ROV surveys (30 m video transects) at 130 sites across 18 reefs throughout the CSMP (Figure 1).

**FIGURE 2:**
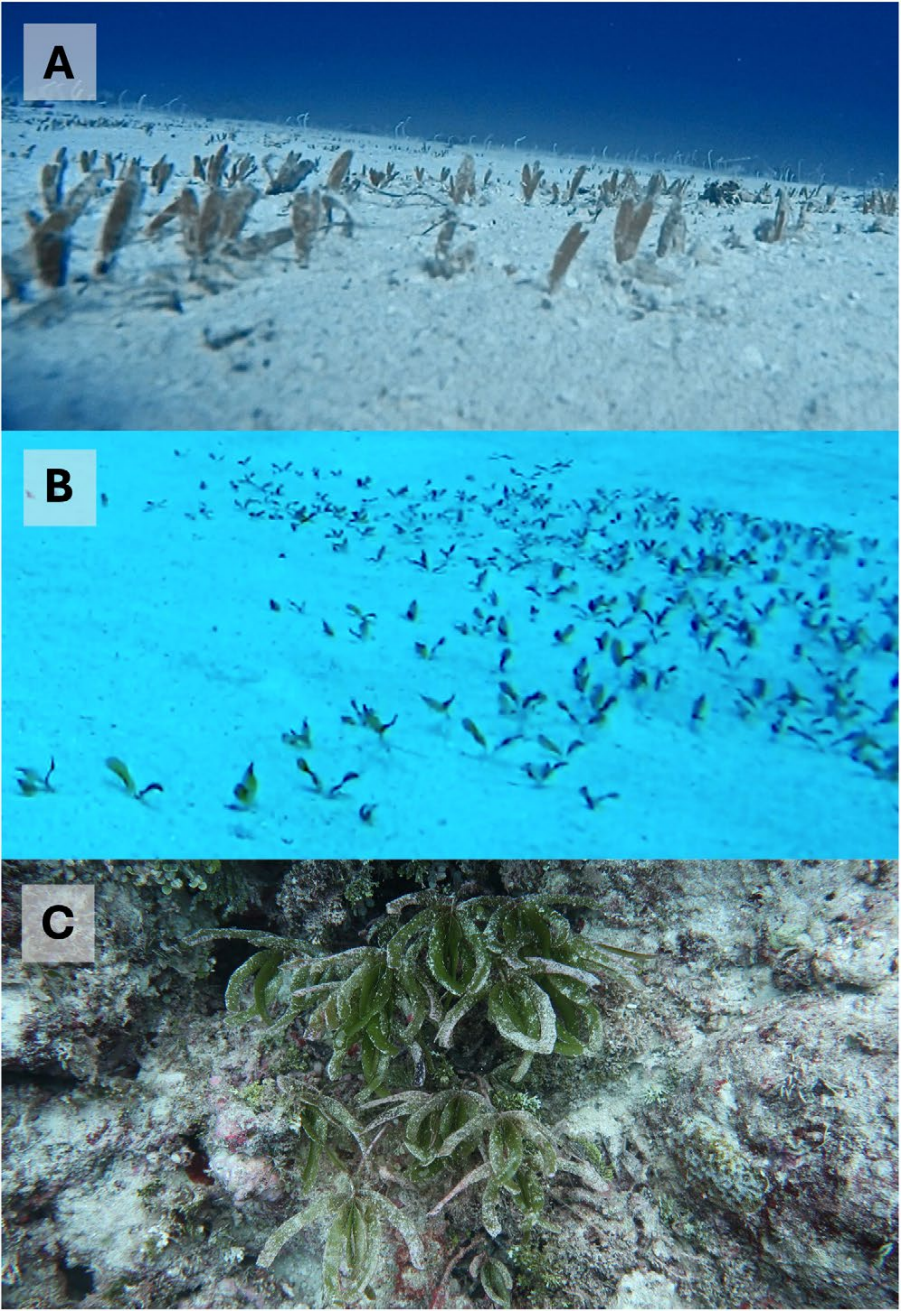
Previous records of relatively small patches of seagrass in the Coral Sea Marine Park *Halophila decipiens* (A) in the lagoon (43 m) and (B) on the outer slope of East Diamond Islet (61 m) and (C) a single patch of *Thalassodendron ciliatum* in between the shallow (< 10 m) reef matrix at Holmes reef (photo credits G. Galbraith and A. Hoey).

Despite this significant survey effort, the occurrence of seagrass in the CSMP is rare and to date, only three species of seagrass have been reported from the CSMP: *Halophila ovalis* , *Halophila decipiens* (Galbraith, Cresswell, et al. 2022; Galbraith et al. 2024; Tol et al. 2023) and *Thalassodendron ciliatum* (Hoey pers. Obs.). Both *H. ovalis* and *H. decipiens* were recorded in the lagoons of the Tregrosse Reefs (Diamond Islets) and Herald Cays in 2022 by Tol et al. (2023) using drop‐cameras and on the leeward outer slopes of East Diamond Islet at 61 m by Galbraith, McClure, et al. (2022) and Galbraith et al. (2024) during ROV surveys. *T. ciliatum* is only known previously from the Holmes Reefs where a small plant was found growing between the reef structure during coral reef health surveys in 2021 (Hoey personal obs). However, the localised distributions of all these previous observations are generally small (< 20m^2^) with sparse density of rhizomes and leaves (Figure 2). This study reports the first and most extensive occurrence of a *Thalassodendron ciliatum* meadow in the southern CSMP, recorded during an exploratory survey from the lagoon of Saumarez Reef. We discuss the unique nature of this observation within the context of the ecological function of seagrass meadows, associated reef fish biodiversity and the requirement for future detailed studies of this site.

## 2 Methods

During ongoing reef health monitoring conducted by James Cook University in the CSMP, ROV surveys were conducted in the lagoon of Saumarez Reef in the southern CSMP (March 2024). The ROV configuration and operation followed the method described in Galbraith, Cresswell, et al. 2022. Briefly, a micro‐ROV (BlueRobotics BlueROV2) was equipped with a high‐definition forward‐facing video camera (GoPro Hero 8) in addition to the onboard navigation camera and three other GoPro cameras (left, right and downward facing), set to capture still images of the underlying and surrounding benthos every 10 s. A submerged coral bommie in the Saumarez lagoon was identified using navigational bathymetric charts (−21.749354, 153.756675) and an opportunistic exploratory ROV dive was conducted on this site.

The ROV was deployed from a small surface vessel and was piloted around the circumference of the base of the bommie (25 m depth). Once the base had been surveyed, the ROV conducted a roving transect (Ajemian et al. 2015) up the side of the bommie until it reached the summit (7 m depth). The ROV then descended from the summit down the other side of the bommie and conducted another roving transect on the descent back to the base. In this way, the majority of the bommie was surveyed by the video and still‐image cameras. A primary objective of the CSMP reef health monitoring program is to quantify reef fish biodiversity. In line with these broader objectives and given the known importance of seagrass meadows for reef fishes (Nagelkerken et al. 2000; Sambrook et al. 2019) we therefore applied the same video analysis methods to quantify the reef fish communities associated with the seagrass meadow as used in our established ROV coral reef surveys (Galbraith, Cresswell, et al. 2022; Hoey et al. 2022, 2023). Footage from the forward‐facing video camera was analysed in a standard video player (VLC media player) and all individual fish entering the field of view were counted and identified to species level. We recorded the habitat in which each individual fish was seen as either seagrass, reef, seagrass/lagoon edge or seagrass/reef edge. To check for the presence of seagrass away from the bommie, the ROV also conducted five transects (50 m length, timed swim estimated distance) perpendicular from the bommie at evenly spaced points around the structure base.

## 3 Results

**FIGURE 3:**
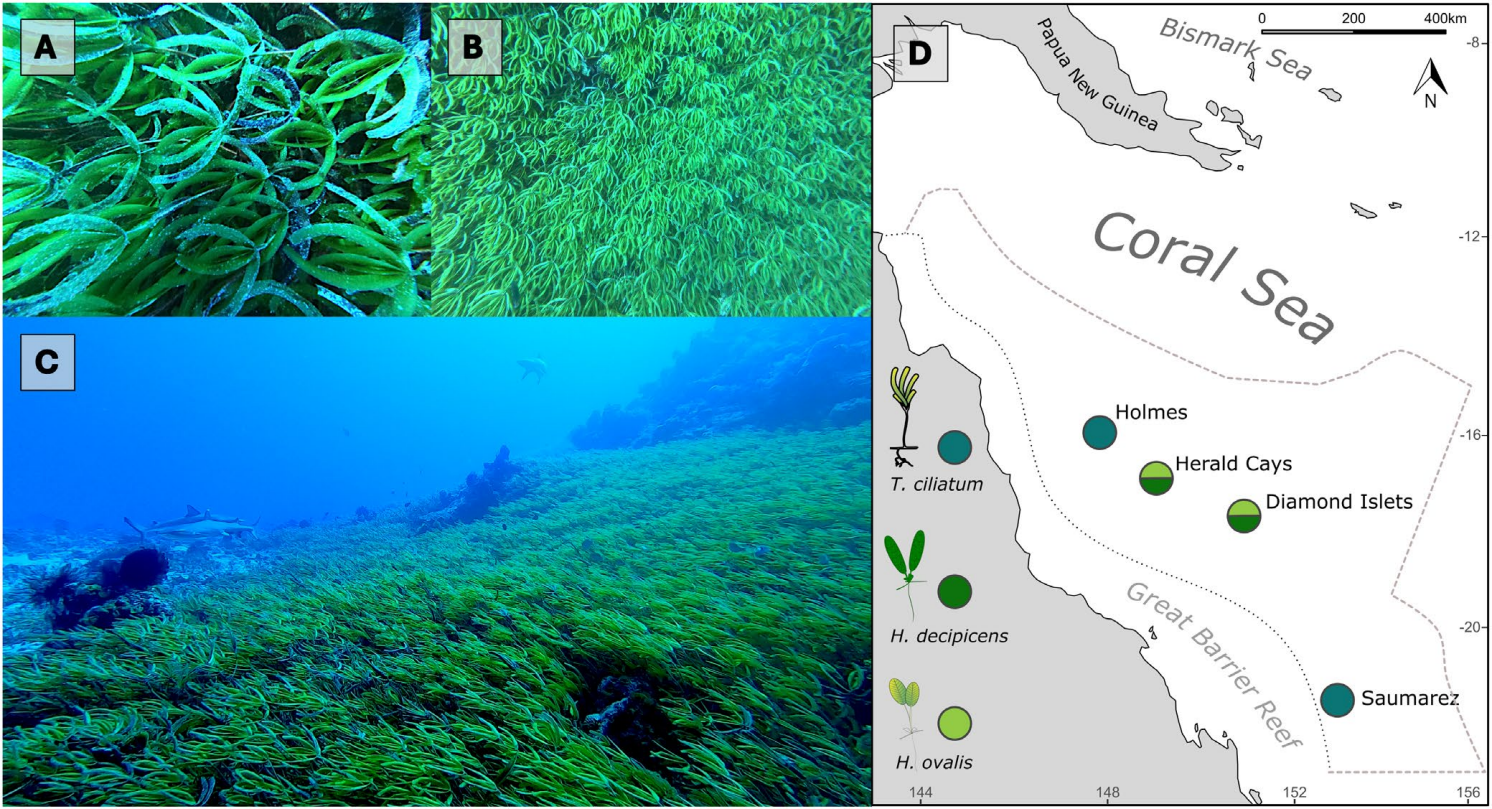
(A–C) Show the dense meadow of *Thalassodendron ciliatum* found at Saumarez Reef in the southern Coral Sea Marine Park, March 2024. (D) Shows the locations of previous seagrass records from the CSMP at Holmes Reefs ( *T. ciliatum* ), Herald Cays (*H. ovalis and H. decipiens*) and Diamond Islets ( *H. ovalis* and *H. decipiens* ). Dashed boundary indicates the extent of Australia's Coral Sea Marine Park and western border with the neighbouring Great Barrier Reef Marine Park.

A dense extensive meadow of *Thalassodendron ciliatum* was recorded around the base of the bommie at a depth of 25m (Figure 3). This is the first record of *T. ciliatum* in the southern CSMP and the most extensive record of seagrass in the CSMP. Based on the known speed (0.2 m.s^−1^) and travelling time of the ROV, we estimate the meadow to cover a band of between 8 and 10 m width around the bommie base, with an outer circumference of approximately 110 m. Using the same ROV speed (0.2 m.s^−1^) we estimate the bommie to have an approximate diameter of 20 m at its base. This equates to a conservative estimated area of ~700 m^2^ seagrass cover (where annulus area = *π* (*R* ^2^ [bommie radius of 10 m + seagrass meadow width of 8 m] − *r* ^2^ [bommie radius 10 m])). There was a distinct boundary between the edge of the seagrass meadow and the surrounding sandy substrate of the lagoon. No further seagrass rhizomes or leaves were observed in photos or video captured by perpendicular transects conducted away from the bommie over the surrounding sandy habitat.

**FIGURE 4:**
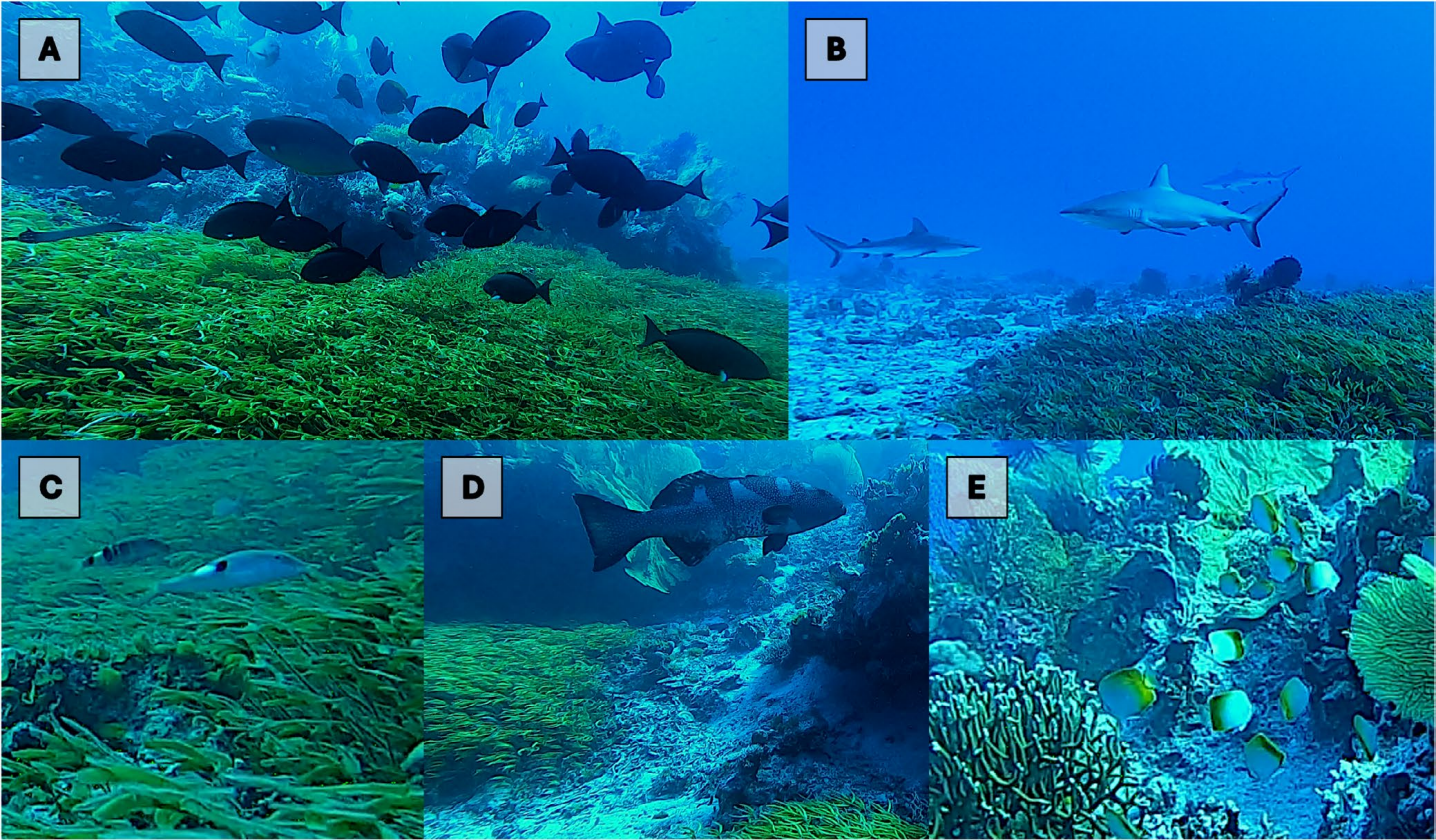
Some of the fish species observed by ROV associated with the seagrass meadow and the bommie (A) Mixed schools of acanthurids and lutjanids (B) *Carcharhinus amblyrhynchos* (C) juvenile *Parupeneus* spp. (D) *Plectropomus laevis* and (E) a school of *Chaetodon guentheri* .

In total, 78 species of fish were recorded in proximity to the seagrass meadow (either within, above or adjacent to) representing 47 genera from 19 families (Table A1). Large predators (e.g., *Plectropomus laevis, Macolor niger, Carcharhinus albimarginatus, C. amblyrhynchos, Triaenodon obesus, Gymnosarda unicolor*) were conspicuous and juvenile fishes from multiple trophic and taxonomic groups were abundant within the seagrass (families Lethrinidae, Mullidae, Siganidae, Labridae, Acanthuridae). There was a notable high abundance of sharks (11 individuals), coral trout (10 large individuals) and butterflyfishes (127 individuals) observed both on the reef structure and around the seagrass meadow (Figure 4).

## 4 Discussion

Unlike the three previous records of seagrass from the CSMP (Hoey pers. obs., Galbraith, Cresswell, et al. 2022, Tol et al. 2023), the observation of *Thalassodendron ciliatum* at Saumarez Reef is a dense meadow, fully covering the underlying substrate with 100% seagrass cover (Figure 3a,b). *T. ciliatum* has a wide global distribution from the Red Sea and South Africa to the Indo‐Pacific (recorded from Palau, the Solomon Islands, Vanuatu and Papua New Guinea), it is absent from most Pacific Island countries (Coles et al. 2003; McKenzie et al. 2021) and is rarely reported from east coast Australia (Lanyon 1986; Collier et al. 2021). This observation represents the most southern occurrence of *T. ciliatum* in the Coral Sea (Figure 3d) which is at a comparable latitude to a 1951 herbarium specimen collected at Stradbroke Island, Queensland (Department of Environment and Natural Resources 2024).

The combination of *T. ciliatum* life‐history traits, with the density and offshore location of the meadow observed in the Saumarez Reef lagoon, suggests this current observation to be a persistent and enduring deep‐water seagrass meadow in the CSMP (Kilminster et al. 2015). *Thalassodendron* spp. are characterised by slow growth, are long‐lived and late maturing. Although generally more resistant to physiological disturbance than fast‐growing species that form transient meadows, *Thalassodendron* spp. do not generate seed banks and are therefore slow to recover from severe disturbances (Collier et al. 2021). Major disturbances in the CSMP include recurrent marine heatwaves and high cyclonic intensity which have significantly impacted some of the CSMP shallow reefs (Hoey et al. 2020, 2023). Like scleractinian corals, seagrasses are negatively affected by the consequences of anthropogenic climate change and excessive wave action associated with severe storms which can cause die‐off and the physical removal of large meadows (Rasheed et al. 2014; Colin 2018; Oprandi et al. 2020). Although deep‐water marine habitats are not immune to global environmental change (Rocha et al. 2018), water depth is considered an important aspect of habitat resilience traits for seagrass meadows (Kilminster et al. 2015). The depth of the Saumarez Reef lagoon (20–40 m throughout) may therefore provide significant shelter from both prevailing and storm‐generated wave action. Indeed, the response of tropical marine systems to ocean heatwaves is modulated by local disturbance regimes in combination with other habitat characteristics like depth and isolation from other anthropogenic stressors (Baum et al. 2023). For coastal seagrasses, the effects of terrestrial runoff and riparian flood plumes are major threats as global intensive use of agricultural fertilisers, pesticides and the frequency and intensity of flooding events increase (Butchart et al. 2010; Turschwell et al. 2021). In both cases, mortality in seagrasses has been linked to declines in water quality (e.g., pollution and turbidity) and other factors which reduce seagrass growth and survival (e.g., burial by sediment and sediment quality) (Orth et al. 2006; Cabaço et al. 2008; Fraser and Kendrick 2017). These anthropogenic land‐based threats to coastal seagrasses therefore likely render isolated offshore seagrass meadows in deep environments important future marine habitats for many marine taxa (Esteban et al. 2018).

The fishes recorded from the seagrass meadow and around the bommie at Saumarez Reef represent 21% of all species recorded to date by shallow reef monitoring surveys in the CSMP (Hoey et al. 2023). Although area‐based surveys of the fish communities associated with the bommie were not conducted (i.e., belt transects), large schools (> 150 individuals) of acanthurids, chaetodontids, caesionids and lutjanids were notable at the site as well as large predators including *Carcharhinus albimarginatus, C. amblyrhynchos and Plectropomus laevis* (Figure 4). Coral bommies and similar isolated patch reef habitats are known to support high densities and species richness of coral reef fishes, particularly predatory species (Letessier et al. 2019; Cresswell et al. 2023) compared to both the surrounding pelagic environment and contiguous fringing reef systems (Galbraith et al. 2021). These patterns are hypothesised to be driven variously by enhanced trophic subsidies focused on the structure by physical processes (Leitner et al. 2021; Galbraith, McClure, et al. 2022; Galbraith et al. 2023), animal‐mediated nutrient transfer (White et al. 2007; Williams et al. 2018) and the provision of habitat in otherwise unsuitable surrounding environments (Fahrig 2013). In the case of the bommie patch reef in this study, we also consider that the combination of seagrass, coral reef and sandy lagoon habitats in close proximity provides multiple habitat functions for a greater number of species (and at different ontogenetic stages) than a single habitat alone (Pianka 1966; Sambrook et al. 2019; Hall and Kingsford 2021). This likely contributes to both the relatively high diversity and large aggregations of reef fishes and predators despite the relatively small area of the study site. The effect of habitat heterogeneity can be further enhanced by edge effects which are known to generate distinct diversity patterns as a result of transitioning conditions between habitats (Murcia 1995) including coral reefs (Sambrook et al. 2019) and seagrass meadows (Dorenbosch et al. 2005).

Many of the fish species observed at the site, particularly juvenile lethrinids and siganids, were only present in the seagrass meadow or directly adjacent on the edge of the seagrass‐sand interface. No other seagrass meadows have been reported from the CSMP or wider offshore Coral Sea; therefore, comparisons within the region are limited. The most similar reported habitat we are aware of is a seagrass meadow surrounding a coral bommie from Green Island, an inshore island 27 km north‐east of Cairns, in the northern Great Barrier Reef (GBR), Australia (Choat and McCormick 1988). Here, the presence of seagrass was also found to affect the distribution and relative abundance of some fish species, in particular a high abundance of juvenile scarids, siganids, lethrinids, lutjanids and mullids within the seagrass meadow but not in the surrounding sand habitat or on the bommie itself. Given the role of coastal seagrass meadows as nursery habitats for juvenile fishes (reviewed by Sambrook et al. 2019), offshore meadows would be expected to provide similar ecological function. However, their importance for settlement and recruitment of some fishes may be greatly enhanced within the CSMP, given that reefs here are significantly spatially isolated from each other and associated non‐reef nursery habitats, unlike the GBR. In addition to valuable ecological functions, high‐density seagrass beds in offshore deep water are also of high conservation value given their isolation from land‐based environmental stressors and depth‐related resilience to the increasing frequency and intensity of storms (Rasheed et al. 2014; Rasheed and Unsworth 2011; Jones et al. 2021). However, like coral reefs, global assessments of seagrass largely focus on shallow‐water coastal habitats, but evidence suggests that deep‐water meadows are both extensive and productive (Rasheed et al. 2014; Coles et al. 2009; York et al. 2015). Further exploration of deep‐water marine habitats is required to refine global seagrass habitat assessments and establish ecological similarities and differences between shallow‐coastal and deep‐offshore seagrass meadows.

Current records of deep‐water seagrasses are almost exclusively found in Australia, suggested to be a product of broad continental shelves with oligotrophic conditions and high water clarity that enable seagrasses to colonise at greater depths (Martin et al. 2023). These conditions are true of the CSMP where high light attenuation extends to mesophotic depths on outer reefs and in reef lagoons (Ceccarelli et al. 2013). Submerged coral bommies, as surveyed by this study, are also ubiquitous within the deep lagoons of the reefs of the CSMP and many possess similar aggregations of predators and high‐density fish communities (Galbraith personal obs). These sites would make effective sampling targets to quantify the amount of additional coral reef habitat available, reef fish biodiversity within the lagoons and to investigate the potential for other seagrass meadows around these structures. That said, scientific diving to the depth of this observation at 25 m is limited by institutional and legislative regulations given the remote nature of the survey site. Future surveys using rapid mobile remote methods like ROVs or towed camera systems that can spend more time underwater at depth and cover larger areas than feasible by diver‐based methods would therefore greatly enhance the likelihood of finding other seagrass meadows at other Coral Sea reefs.

The observation of an extensive and dense meadow of *T. ciliatum* reported here represents new knowledge of the spatial distribution of seagrasses in the CSMP, and the Coral Sea and western‐Pacific more broadly. Meadows such as these are likely of high ecological importance for the offshore reefs, which are spatially isolated from other offshore reefs and other major reef systems like the adjacent Great Barrier Reef. Though we present here a single observation, the persistence of the meadow discovered by this study should be assessed over time, alongside ongoing reef health monitoring surveys. Further surveys within CSMP reef lagoons are required to establish the full extent of deep‐water seagrass in the region and to conduct more detailed studies of seagrass density, connectivity with adjacent habitats and biodiversity assessments of other reef taxa (e.g., invertebrates and benthic organisms). Given the historic lack of research attention directed at deeper reef and non‐reef habitats, the diversity of habitat configurations in the lagoons of these reefs is likely much greater than is currently known. It is important to continue or enhance protection of Saumarez Reef, and any future areas discovered to have seagrass in the CSMP, from anthropogenic habitat disturbances.

## Author Contributions

**G. F. Galbraith:** conceptualization (equal), data curation (lead), formal analysis (lead), funding acquisition (equal), investigation (equal), methodology (equal), visualization (lead), writing – original draft (lead), writing – review and editing (equal). **B. J. Cresswell:** conceptualization (equal), data curation (supporting), formal analysis (supporting), investigation (equal), methodology (equal), writing – review and editing (equal). **M. Russell:** conceptualization (equal), project administration (supporting), writing – review and editing (equal). **A. S. Hoey:** conceptualization (equal), funding acquisition (lead), project administration (lead), resources (lead), writing – review and editing (equal).

## Conflicts of Interest

The authors declare no conflicts of interest.

## Data Availability

All data analysed from ROV survey footage are presented in this article.

## Appendix A

**TABLE A1:** List of fish species observed within or above the seagrass meadow or associated with the adjacent bommie structure (presence in each habitat denoted by X).

Family	Genus	Species	Lagoon/Seagrass Edge	Seagrass	Reef/Seagrass Edge	Reef
Acanthuridae	*Acanthurus*	*albipectoralis*		X	X	X
Acanthuridae	*Acanthurus*	*dussumieri*		X	X	X
Acanthuridae	*Acanthurus*	*pyroferus*		X	X	X
Acanthuridae	*Ctenochaetus*	*binotatus*				X
Acanthuridae	*Ctenochaetus*	*striatus*			X	X
Acanthuridae	*Naso*	*hexacanthus*		X	X	
Acanthuridae	*Naso*	*brevirostris*		X	X	
Acanthuridae	*Naso*	*litteratus*		X	X	X
Acanthuridae	*Naso*	*vlamingii*	X	X		
Acanthuridae	*Zebrasoma*	*velifer*			X	X
Acanthuridae	*Zebrasoma*	*scopas*		X	X	
Caesionidae	*Caesio*	*tile*	X			X
Caesionidae	*Pterocaesio*	*marri*	X			X
Carcharhinidae	*Triaenodon*	*obesus*	X	X	X	
Carcharhinidae	*Carcarhinus*	*amblyrhynchos*	X	X		
Chaetodontidae	*Chaetodon*	*plebeius*		X	X	X
Chaetodontidae	*Chaetodon*	*kleinii*			X	X
Chaetodontidae	*Chaetodon*	*guentheri*				X
Chaetodontidae	*Chaetodon*	*unimaculatus*		X	X	
Chaetodontidae	*Chaetodon*	*pelewensis*			X	X
Chaetodontidae	*Chaetodon*	*lunulatus*		X	X	X
Chaetodontidae	*Chaetodon*	*lineolatus*		X	X	X
Chaetodontidae	*Chaetodon*	*mertensii*			X	X
Chaetodontidae	*Forcipiger*	*flavissimus*		X	X	X
Chaetodontidae	*Hemitaurichthys*	*polylepis*				X
Cirrhitidae	*Paracirrhites*	*arcatus*			X	X
Cirrhitidae	*Paracirrhites*	*forsteri*			X	X
Ephippidae	*Platax*	*teira*		X	X	
Labridae	*Anampses*	*femininus*			X	X
Labridae	*Bodianus*	*axillaris*			X	
Labridae	*Cheilinus*	*trilobatus*			X	
Labridae	*Cheilinus*	*oxycephalus*			X	X
Labridae	*Cirrhilabrus*	*punctatus*				X
Labridae	*Halichoeres*	*hortulanus*		X	X	X
Labridae	*Cheilio*	*inermis*	X	X		
Labridae	*Labrichthys*	*unilineatus*			X	
Labridae	*Labroides*	*bicolor*		X	X	X
Labridae	*Labroides*	*dimidiatus*		X	X	X
Labridae	*Pseudocheilinus*	*hexataenia*			X	X
Lethrinidae	*Lethrinus*	*nebulosus*	X	X		
Lethrinidae	*Lethrinus*	*atkinsoni*	X	X		
Lethrinidae	*Monotaxis*	*grandoculis*		X		
Lutjanidae	*Lutjanus*	*bohar*	X			X
Lutjanidae	*Macolor*	*niger*	X	X	X	X
Mullidae	*Parupeneus*	*multifasciatus*	X	X		
Mullidae	*Parupeneus*	*pleurostigma*	X	X		
Nemipteridae	*Scolopsis*	*bilineata*	X	X	X	
Pomacanthidae	*Centropyge*	*bispinosa*			X	X
Pomacanthidae	*Pygoplites*	*diacanthus*			X	X
Pomacentridae	*Acanthochromis*	*polyacanthus*			X	X
Pomacentridae	*Amblyglyphidodon*	*aureus*			X	X
Pomacentridae	*Amblyglyphidodon*	*leucogaster*			X	X
Pomacentridae	*Amblyglyphidodon*	*curacao*				X
Pomacentridae	*Chromis*	*iomelas*			X	X
Pomacentridae	*Chromis*	*ternatensis*			X	X
Pomacentridae	*Chromis*	*lepidogenys*				X
Pomacentridae	*Chromis*	*xanthura*			X	X
Pomacentridae	*Dascyllus*	*reticulatus*		X	X	
Pomacentridae	*Neoglyphidodon*	*nigroris*			X	X
Pomacentridae	*Plectroglyphidodon*	*lacrymatus*			X	X
Pomacentridae	*Pomacanthus*	*brachialis*			X	X
Scaridae	*Chlorurus*	*spilurus*		X	X	X
Scaridae	*Scarus*	*frenatus*		X	X	
Scaridae	*Scarus*	*oviceps*		X	X	X
Scaridae	*Scarus*	*niger*			X	X
Scaridae	*Scarus*	*forsteni*		X	X	X
Scaridae	*Scarus*	*chameleon*		X	X	
Scaridae	*Thalassoma*	*lunare*		X	X	X
Scaridae	*Thalassoma*	*hardwicke*		X	X	X
Scombridae	*Gymnosarda*	*unicolor*	X			
Serranidae	*Cephalopholis*	*cyanostigma*				X
Serranidae	*Cephalopholis*	*leopardus*				X
Serranidae	*Plectropomus*	*laevis*		X	X	
Serranidae	*Pseudanthias*	*pictilis*			X	X
Siganidae	*Siganus*	*woodlandi*	X	X		
Tetraodontidae	*Arothron*	*hispidus*		X	X	
Zanclidae	*Zanclus*	*cornutus*		X		X
